# Protocol for pilot cluster RCT of project respect: a school-based intervention to prevent dating and relationship violence and address health inequalities among young people

**DOI:** 10.1186/s40814-019-0391-z

**Published:** 2019-01-22

**Authors:** Rebecca Meiksin, Elizabeth Allen, Joanna Crichton, Gemma S. Morgan, Christine Barter, Diana Elbourne, Kate Hunt, G. J. Melendez-Torres, Steve Morris, H. Luz Mc Naughton Reyes, Joanna Sturgess, Bruce Taylor, Honor Young, Rona Campbell, Chris Bonell

**Affiliations:** 1 London School of Hygiene & Tropical Medicine, Keppel Street, London, WC1E 7HT UK; 2 University of Bristol, 39 Whatley Road, Bristol, BS8 2PS UK; 3 University of Central Lancashire, Preston, Lancashire PR1 2HE UK; 4 University of Stirling, Stirling, FK9 4LA UK; 5 Cardiff University, 1-3 Museum Place, Cardiff, CF10 3BD UK; 6 University College London, 1-19 Torrington Place, London, WC1E 7HB UK; 7 University of North Carolina, 319 G Rosenau Hall, Chapel Hill, NC 27599 USA; 8 NORC at the University of Chicago, 4350 East West Highway, Room 733, Bethesda, MD 20814 USA

**Keywords:** Dating and relationship violence, Violence prevention, School intervention, Cluster randomised trial, Realist evaluation, Process evaluation, Adolescent

## Abstract

**Background:**

Dating and relationship violence (DRV)—intimate partner violence during adolescence—encompasses physical, sexual and emotional abuse. DRV is associated with a range of adverse health outcomes including injuries, sexually transmitted infections, adolescent pregnancy and mental health issues. Experiencing DRV also predicts both victimisation and perpetration of partner violence in adulthood.

Prevention targeting early adolescence is important because this is when dating behaviours begin, behavioural norms become established and DRV starts to manifest. Despite high rates of DRV victimisation in England, from 22 to 48% among girls and 12 to 27% among boys ages 14–17 who report intimate relationships, no RCTs of DRV prevention programmes have taken place in the UK. Informed by two school-based interventions that have shown promising results in RCTs in the USA—Safe Dates and Shifting Boundaries—Project Respect aims to optimise and pilot a DRV prevention programme for secondary schools in England.

**Methods:**

Design: optimisation and pilot cluster RCT. Trial will include a process evaluation and assess the feasibility of conducting a phase III RCT with embedded economic evaluation. Cognitive interviewing will inform survey development.

Participants: optimisation involves four schools and pilot RCT involves six (four intervention, two control). All are secondary schools in England. Baseline surveys conducted with students in years 8 and 9 (ages 12–14). Follow-up surveys conducted with the same cohort, 16 months post-baseline. Optimisation sessions to inform intervention and research methods will involve consultations with stakeholders, including young people.

Intervention: school staff training, including guidance on reviewing school policies and addressing ‘hotspots’ for DRV and gender-based harassment; information for parents; informing students of a help-seeking app; and a classroom curriculum for students in years 9 and 10, including a student-led campaign.

Primary outcome: the primary outcome of the pilot RCT will be whether progression to a phase III RCT is justified. Testing within the pilot will also determine which of two existing scales is optimal for assessing DRV victimisation and perpetration in a phase III RCT.

**Discussion:**

This will be the first RCT of an intervention to prevent DRV in the UK. If findings indicate feasibility and acceptability, we will undertake planning for a phase III RCT of effectiveness.

**Trial registration:**

ISRCTN, ISRCTN 65324176. Registered 8 June 2017.

**Electronic supplementary material:**

The online version of this article (10.1186/s40814-019-0391-z) contains supplementary material, which is available to authorized users.

## Background

### Dating violence and public health

Dating and relationship violence (DRV)—used to describe intimate partner violence during adolescence [1–3]—encompasses threats, emotional abuse, controlling behaviours, physical violence and coerced, non-consensual or abusive sexual activities perpetrated by a partner [4]. Globally, 30% of ever-partnered women report violence from current or previous partners at some point in their lives [5, 6]. Evidence suggests that partner violence begins early, with prevalence of DRV victimisation already reaching 29.4% among girls ages 15–19 [6–10]. Norms accepting of gender-based violence and harassment strongly correlate with DRV perpetration and victimisation [9–13] and young people identify concerns about social repercussions as a barrier to intervening in DRV as a bystander [14]. Young people who experience DRV are more likely to be victims or perpetrators of relationship violence as adults [15–17]. Early experience of DRV is also associated with subsequent adverse outcomes such as substance misuse and anti-social behaviour [18–20], sexually transmitted infections (STIs) and teenage pregnancy [21], eating disorders [17], suicidal behaviours and other mental health problems [17, 22], physical injuries [23] and low educational attainment [22]. Experiencing violence during pregnancy correlates with poorer maternal and neonatal health outcomes [21, 24]. In addition to its harms, domestic violence is associated with significant financial costs to health systems. In 2008 in the UK, it was estimated that domestic violence cost the National Health Service £1.73 billion per year with total costs to the UK economy of £15.73 billion per year [25].

### Rationale for proposed study

There is a pressing need to prevent DRV in the UK. Recent surveys of English young people suggest victimisation prevalence of 22–48% for young women and 12–27% for young men aged 14–17 years who report an intimate relationship [26–28]. Universal, primary prevention of DRV is required since these behaviours are widespread and under-reported [29]. Prevention during early adolescence is important because this is the time when dating behaviours begin, behavioural norms become established and DRV starts to manifest [30, 31]. Schools are a key site to achieve this since they are settings in which young people are socialised into gender norms and in which significant amounts of gender-based harassment and DRV go unchallenged [32, 33]. Because DRV arises not only from individual deficits in communication and anger management skills [34] but also from sexist gender norms and pervasive gender-based harassment [23, 35–37], within schools multi-component interventions—for example, addressing school curricula, policies and environments—are required [38] to address factors driving DRV at multiple levels of the social ecology.

There is thus a pressing need for a UK-based randomised controlled trial (RCT) of a universal multi-component, school-based prevention intervention, informed by existing evidence, which targets early adolescents. Project Respect aims to meet this need. The Project Respect intervention is designed to address similar topics to those targeted by the effective Safe Dates [39] and Shifting Boundaries interventions [40]. The programme’s theory of change outlines hypothesised pathways to programme outcomes. There is a need for a UK-specific intervention because given cultural differences, direct replication of a US intervention is unlikely to be effective in the UK [41]. We will therefore begin by working with UK secondary school staff and students to elaborate and optimise the intervention and produce the manual, curriculum and other intervention materials. We will then subject Project Respect to a pilot cluster RCT to assess feasibility and acceptability and optimise methods prior to a phase III RCT. This will be the first UK RCT of an intervention to prevent DRV among young people.

### Interventions

Guidance on domestic violence published by the UK National Institute for Health and Care Excellence in 2014 has highlighted the lack of current evidence for interventions preventing adolescent DRV [42]. Recent Cochrane and Campbell reviews of DRV prevention have conducted meta-analyses to estimate effects on behavioural, attitudinal and knowledge outcomes, finding overall effects on knowledge and attitude, but not behaviour [43, 44]. However, more promising results for behaviour are reported from RCTs of the Safe Dates and Shifting Boundaries interventions [39, 40]. These were included in the Campbell but excluded from the Cochrane review; exclusion of Safe Dates and Shifting Boundaries from the Cochrane review was due to incomplete reporting and recent publication respectively. The authors of the Cochrane review noted that non-inclusion of Safe Dates was a major limitation of their review. These interventions were also identified in a broader review of interventions to prevent sexual violence perpetration as the only effective interventions addressing this issue among young people [45].

The Safe Dates curriculum was delivered over ten sessions to eighth and ninth grade students (aged 13–15 years) in North Carolina, USA and focused on the consequences of DRV, gender roles, conflict management skills, norms, help-seeking and student participation in drama and poster activities. A school cluster RCT [39, 46] reported significantly reduced perpetration of physical DRV and victimisation of serious physical DRV (*p* < 0.05 for both) and significantly reduced perpetration and victimisation of sexual DRV (*p* = 0.04, *p* = 0.01 respectively) at 4-year follow-up. The duration of these effects suggests these might be real behavioural effects rather than merely social desirability effects on reporting. The intervention was equally effective for females and males [47].

A four-arm school cluster RCT of the Shifting Boundaries interventions allocated schools to receive one of the following: curriculum intervention, school environment intervention, combined intervention and neither intervention [40]. The curriculum comprised six sessions on the consequences of DRV, the social construction of gender roles and what constitutes healthy relationships. The environment intervention included higher levels of staff presence in hot-spots for gender-based harassment mapped by students, including use of joint faculty and student safety committees to help guide the placement of security personal, posters and increased sanctions for perpetrators including use of building-based temporary restraining orders and use of joint faculty-student safety committees. The environment-only and the combined interventions were effective in reducing sexual violence victimisation at 6-months follow-up (respectively OR = 0.662 *p* = 0.028; OR = 0.659 *p* = 0.011). There were also reductions in sexual violence perpetration in the environment-only and combined intervention (respectively OR = 0.527 *p* = 0.002; OR = 0.524 *p* = 0.001). There was no evidence of these effects with the curriculum-only intervention. Results show similar benefits for females and males and for those with and without a history of DRV [48]. The Cochrane review recommended that further research on multi-component interventions in schools is a priority. The Campbell review recommended that future interventions more explicitly address skills and the role of peer norms in preventing DRV.

### Benefits and risks

There are major potential public health benefits arising from the prevention of adolescent DRV, which affects a substantial proportion of young people in the UK. Components of the Project Respect intervention are similar to those comprising the effective Safe Dates and Shifting Boundaries interventions, which do not report physical or psychological harm stemming from such an intervention blending structural and curriculum components. Evidence suggests DRV research is unlikely to pose psychological risks to research participants [49]. Research participants will be informed that their participation in the research is voluntary and that they may withdraw at any point. As we cannot be certain prior to piloting that this intervention research poses no risk to participants, our process evaluation will explore potential for harm. Any potential mechanisms of harmful effects of the intervention will be explored through qualitative data in this pilot RCT and in later evaluation phases. We will closely liaise with participating schools to facilitate data collection with students. We will minimise disruption for staff and ensure student privacy and confidentiality both by employing strategies used successfully in our past work, such as having the trial manager liaise directly with each participating school to identify convenient times and places for data collection, and by piloting innovative methods in this context, such as the use of computer assisted self-interview (CASI) surveys. Ethical issues are discussed in more detail below.

## Methods

### Research aims, research questions and objectives

#### Aims

I. With stakeholders, to elaborate and optimise Project Respect, informed by existing research.
II. To conduct a pilot RCT (four intervention, two control schools) in southern England.

#### Research questions

1. Is progression to a phase III RCT justified in terms of pre-specified criteria? These criteria are: randomisation occurs and four or more schools out of six accept randomisation and continue within the study; the intervention is implemented with fidelity in at least three of the four intervention schools; the process evaluation indicates the intervention is acceptable to 70% or more of year 9 and 10 students and staff involved in implementation; CASI surveys of students are acceptable and achieve response rates of at least 80% in four or more schools; and methods for economic evaluation in a phase III RCT are feasible.
2. Which of two existing scales—the Safe Dates (SD) and the short Conflict in Adolescent Dating Relationships Inventory (CADRI-s)—is optimal for assessing DRV victimisation and perpetration as primary outcomes in a phase III RCT, judged in terms of completion, inter-item reliability and fit?
3. What are likely response rates in a phase III RCT?
4. Do the estimates of prevalence and intra-cluster correlation coefficient (ICC) of DRV derived from the literature look similar to those found in the UK so that they may inform a sample size calculation for a phase III RCT?
5. Are secondary outcome and covariate measures reliable and what refinements are suggested?
6. What refinements to the intervention are suggested by the process evaluation?
7. What do qualitative data suggest about how contextual factors might influence implementation, receipt or mechanisms of action?
8. Do qualitative data suggest any potential harms and how might these be reduced?
9. What sexual health and violence-related activities occur in and around control schools?

#### Objectives

**Fig. 1:**
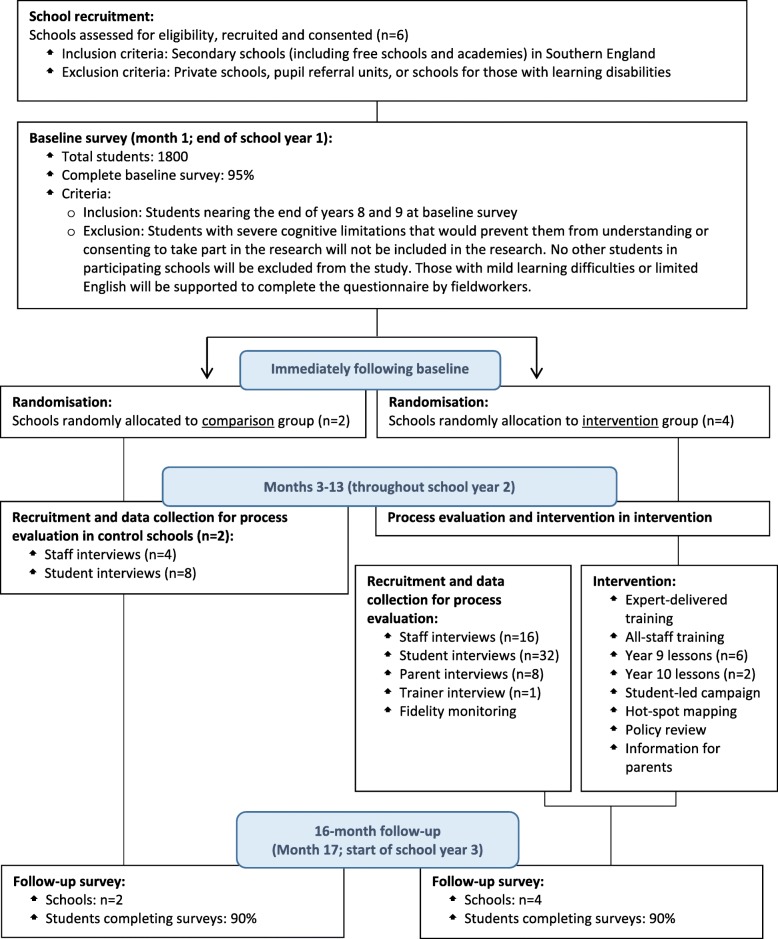
SPIRIT figure for pilot cluster randomised controlled trial of Project Respect

- To elaborate and optimise Project Respect and produce intervention materials in collaboration with the National Society for the Prevention of Cruelty to Children (NSPCC), four secondary schools, youth and policy stakeholders and the originators of effective US programmes informing our intervention.
- To adapt and cognitively test the SD and CADRI-s scales prior to piloting.
- To recruit six schools, undertake baseline CASI survey of two cohorts of students at the end of years 8 and 9 respectively plus online staff survey, and randomise four schools to receive the intervention and two to be usual-treatment controls (see Fig. 1).
- To ensure Project Respect is implemented for students in years 9 and 10, conduct process evaluation, and follow-up student CASI and staff online surveys 16 months post-baseline (start of years 10 and 11).
- To address the above research questions to inform progression to a phase III RCT.

### Research design

#### Intervention elaboration and optimisation and cognitive interviewing to refine DRV scales

The core components of the intervention and the underlying theory of change have been informed by existing research, including studies on the Safe Dates and Shifting Boundaries interventions and existing systematic reviews as described above. Further work is required to elaborate the intervention methods and produce materials (manual, staff training and student curriculum), optimising these for use in the UK. This process will be led by the investigators and NSPCC working in close collaboration, and with the participation of students and teachers drawn from four secondary schools (different to those that will be involved in the pilot RCT), as well as the Advice Leading to Public Health Advancement (ALPHA) group [50]—a young people’s research advisory group—and policy stakeholders. We will elaborate and optimise the intervention through a systematic process involving review by researchers and NSPCC of existing systematic reviews and evaluation reports, elaboration of Project Respect methods and production of draft materials by NSPCC staff and the research team, consultation with stakeholders on the draft intervention materials via two facilitated workshops and web-based consultation and refinement of the draft intervention materials based on feedback. At the same time, we will adapt two existing DRV scales and refine the adaptations by conducting cognitive interviews with young people who are the same age as intended respondents. In cognitive interviewing, a qualitative method for pre-testing and improving survey questions, the focus is on the cognitive processes respondents use to answer survey items [51]. It aims to assess whether survey items are appropriate for their intended purpose [52], and we will use this approach to identify problems respondents encounter with survey items and to assess whether participants understand these items as intended. After adaptation, we will test these two scales in the pilot cluster RCT in order to determine which would be optimal for measuring DRV victimisation and perpetration as the primary outcomes in a phase III RCT. In these cognitive interviews, we will also pre-test selected items on attitudes and norms related to gender and DRV. Cognitive interviewing will occur in one of the schools taking part in elaborating the intervention and will involve eight male and eight female students. Students will complete paper questionnaires covering basic socio-demographics followed by the two DRV scales. They will then be interviewed and asked to ‘think aloud’ about how they answered the questions [53] with some probing [54] about comprehension, recall, judgement and response in relation to selected items [55].

#### Pilot RCT

We will then conduct a pilot cluster RCT (four intervention, two control schools; different to those involved in intervention elaboration and any subsequent phase III RCT), with an integral process evaluation and an embedded economic evaluation feasibility study. The research and intervention teams will be separately managed to ensure the evaluation is independent and that the proposed research does not distort intervention delivery. Although in the phase III RCT the intervention would be delivered over two academic years (targeting a single cohort of students progressing from year 9 to year 10 in this period), in this pilot the intervention will be implemented during one school year to two groups of students, those in year 9 and those in year 10. Curriculum lessons designed for each of these year groups will be piloted with the appropriate year group. One year of piloting is sufficient to assess feasibility and acceptability in order to address our research questions. Similarly, although a future phase III RCT would involve follow-up surveys at 28 months post-baseline, follow-up surveys in the pilot RCT will occur 16 months post-baseline. This timescale is sufficient to assess the feasibility of trial methods among participants of the same age as participants would be in a phase III trial at 28 months. Due to the sensitive nature of the baseline and follow-up student surveys, we will use a repeat cross-sectional rather than longitudinal design. The follow-up surveys will be conducted with the same two cohorts of students who took part in the baseline survey, but surveys will not be linked at the level of the individual. This design does not require that we link respondent names to the responses they submit, therefore protecting students’ anonymity.

The Standard Protocol Items: Recommendations for Intervention Trials (SPIRIT) figure (Fig. 1) outlines the key phases of the study. We provide a SPIRIT checklist in Additional file 1 [56, 57].

### Setting

The Project Respect intervention is intended for all mainstream secondary schools. There is no clear evidence that DRV among UK adolescents is associated with individual socio-economic status (SES) or school-level deprivation [27, 58]. Evaluating Project Respect in a sample of schools over-representing those in deprived areas would therefore unnecessarily undermine the generalisability of our findings.

#### Pilot trial inclusion criteria

- Secondary schools (including free schools and academies) in southern England.

#### Pilot trial exclusion criteria

- Private schools, PRUs and schools designed especially for students with learning disabilities.

### Population

As with similar previous studies [39, 40], Project Respect is a universal intervention for female and male students aged 13–15 years (in years 9 and 10 in UK schools). This age group is appropriate because this is the time when most dating behaviours begin, behavioural norms become established and DRV starts to manifest [30, 31]. Stakeholder consultations suggest provision to year 11 students is not feasible due to UK school exam timetables. In the pilot RCT, the intervention will run for 1 year only, targeting year 9 and 10 students, so that we may assess the intervention feasibility and acceptability.

#### Pilot trial inclusion criteria

- Students nearing the end of years 8 and 9 at the time of the baseline survey

#### Pilot trial exclusion criteria

- Students with severe cognitive limitations that would prevent them from understanding or consenting to take part in the research will not be included in the research. No other students in participating schools will be excluded from the study. Fieldworkers will support students who have mild learning difficulties or limited English proficiency to complete the questionnaire.

### Analytic sample and proposed sample size

The pilot RCT will focus on feasibility and no power calculation for this has been performed. Four schools implementing the intervention in the pilot trial balances the need to assess implementation in a diversity of schools while ensuring the pilot is small enough to be appropriate as a preliminary to a larger phase III RCT. The analytic sample for outcome assessment in the pilot will be a minimum of 1800 students at the ends of years 8 and 9 (aged 12/13 and 13/14 years) at baseline, with follow-up at 16 months. Data on fidelity and acceptability are intended to provide site-specific descriptive estimates rather than to be generalizable to a broader group of schools.

### Recruitment and randomisation

Four schools will be involved in intervention elaboration and optimisation, purposively sampled to vary by region and deprivation (as measured by the income deprivation affecting children index, IDACI). In the subsequent pilot RCT phase, three schools in southeast England and three schools in southwest England will be recruited; these schools will be different from those participating in optimisation. Schools taking part in the pilot RCT will be purposively sampled to ensure variation by deprivation and school-level value-added academic attainment, as approximate indicators of school capacity to deliver Project Respect.

We will recruit schools via letters and telephone calls to schools, local authorities, academy chains and school networks. Response rates will be recorded, as will any stated reasons for non-participation. After baseline CASI surveys with students at the end of years 8 and 9, schools will be stratified by region and randomly allocated 2:1 to intervention/control by the London School of Hygiene and Tropical Medicine (LSHTM) clinical trials unit (CTU). The 2:1 allocation will enable us to pilot randomisation while ensuring sufficient diversity among four schools for piloting the intervention. Retention of control schools will be maximised via £500 payment and feedback of survey data.

### Planned intervention

#### Intervention components

Project Respect is a manualised, multi-component school-based universal prevention intervention.

**Fig. 2:**
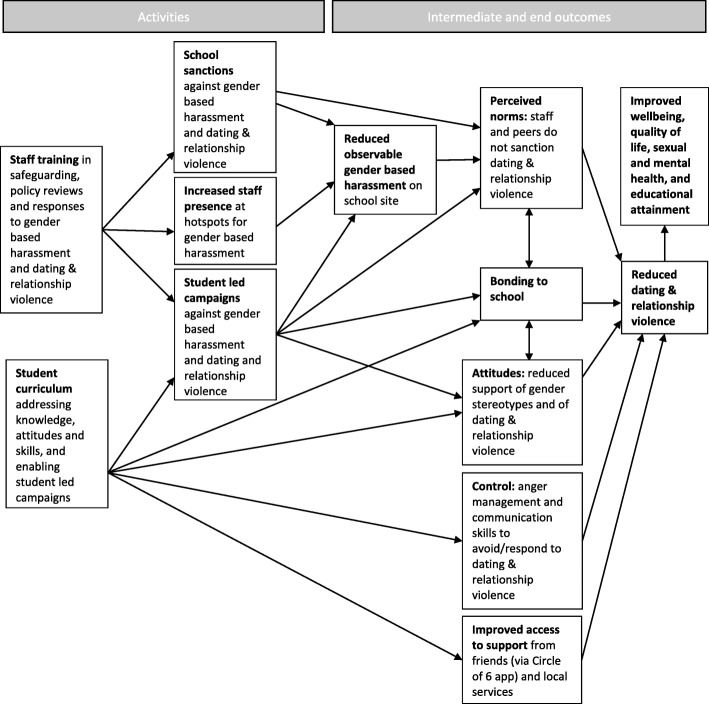
Theory of change for Project Respect

Table 1 summarises the Project Respect intervention according to the items included in the ‘Template for Intervention Description and Replication’ (TIDieR) checklist [59], and Fig. 2 presents the intervention's theory of change.

**Table 1:** Description of the Project Respect intervention using TIDieR checklist items

TIDieR Item	Information on Project Respect intervention
Brief name	Project Respect
Why	We present the theory of change for Project Respect in Fig. 2. The intervention is underpinned by the theory of planned behaviour [93] and the social development model [94]. It is also supported by reviews which suggest that DRV interventions should challenge attitudes and perceived norms concerning gender stereotypes and violence as well as support the development of skills and control over behaviour [38]. Informed by the theory of planned behaviour, Project Respect will aim to reduce DRV by challenging student attitudes and perceived social norms about gender, appropriate behaviour in relationships, and violence; and by promoting student sense of control over their own behaviour. A key element of our theory of change is that attitudes and norms will be challenged not only via the student curriculum but also via actions at the level of the school environment to reduce gender-based harassment observable on the school site and increase school sanctions against gender-based harassment and DRV. Sense of control over behaviour will be promoted via the curriculum components focusing on communication and anger management skills. Informed by the social development model, Project Respect will enable student participation in curriculum lessons and leadership of campaigns in order to maximise learning, increase student bonding to school, and increase acceptance of school behavioural norms. The curriculum also aims to reduce DRV by promoting awareness of the Circle of 6 app [95] and local services, increasing the ability of those who experience DRV to seek support. Project Respect, like the earlier Shifting Boundaries intervention [40], includes a curriculum as well as school-elements. Informed by Shifting Boundaries, the Project Respect curriculum addresses gender roles and healthy relationships and uses hotspot mapping to inform changes in staff patrols of school premises. Informed by the earlier Safe Dates intervention [96], which is primarily curriculum-based, the Project Respect curriculum includes a focus on gender roles, conflict management skills, norms, and help-seeking and incorporates a student-led campaign component.
What materials	Schools allocated to receive the intervention will be provided with various resources. Schools will receive a manual to guide delivery of the intervention. School staff will be offered training (see below) and participants will receive slides to guide delivery of an all-staff training they deliver. Parents of students will be given written information on the intervention and advice on preventing and responding to DRV. Students will be given the opportunity to download the ‘Circle of 6’ app which helps individuals contact friends or the police if threatened by/experiencing DRV. Schools will be provided with written lesson plans and slides to guide delivery of a classroom social and emotional skills curriculum targeting students aged 13–15 years which includes a student-led campaign element.
What procedures	Project Respect is a multi-component school-based universal prevention intervention. The intervention aims to address DRV perpetrated by young people of all genders in heterosexual or same-sex relationships. School policies and rules will be rewritten to ensure that they aim to prevent and respond to DRV and gender-based harassment. Areas on the school site that are identified through student and staff mapping exercises as ‘hotspots’ for DRV and gender-based harassment will be patrolled by staff to prevent and respond to incidents. Responses will include appropriate sanctions for perpetration, support for victims and referral of victims or perpetrators to specialist services where necessary. The curriculum will include lessons that focus on (1) challenging gender norms; (2) defining healthy relationships; (3) inter-personal boundaries, consent, and mapping ‘hotspots’ for gender-based harassment and DRV on the school site; (4) how students can help a friend they are worried about, and empowering students to run campaigns challenging gender-based harassment and DRV; (5) communication and anger management skills relating to relationships; and (6) accessing local services relating to DRV and reviewing student-led campaign ideas. Learning activities will include: information provision; whole class discussions; video vignettes to help students identify abusive behaviours and relationships; quizzes; role plays and exercises; and cooperative planning and review of student-led campaigns. Schools that are randomly allocated to the intervention will be asked to continue with usual provision in addition to implementing the Project Respect intervention.
Who provides	School staff will implement the intervention with support from the NPSCC. Training will be provided by NSPCC for senior leadership and other key school staff to enable them to plan and deliver the intervention in their schools and review school rules and policies to help prevent and respond to DRV and gender-based harassment, and increase staff presence in ‘hotspots’ for these behaviours. Training will then be provided by these trained school staff for all other school staff in safeguarding to prevent, recognise and respond to gender-based harassment and DRV. The NSPCC will further support intervention delivery by offering advice sessions of up to one hour per week to intervention schools.
How	All intervention components will be delivered face-to-face and at the group level.
Where	All components will be delivered on school premises.
When and how much	Training by NSPCC will be provided in a 2–3-h session. Training within the school will be provided in a 60–90-min session. Policy review and hotspot mapping will occur in one or more school management meetings. School patrols will occur throughout the school year. The intervention curriculum will comprise six sessions in year 9 and two booster sessions for the same cohort in year 10, a relatively small number of lessons both years to ensure that the curriculum can be implemented in busy school timetables. As described in the ‘Research design’ section above, lessons in this pilot study will be delivered to students in years 9 and 10 during the same school year rather than to the same cohort over two years.
Tailoring	The intervention will not be tailored.
How well (planned fidelity assessment)	As described in the ‘Process evaluation’ section below, fidelity will be assessed via audio-recordings of the NSPCC-delivered and all-staff trainings, logbooks completed by teaching staff delivering curriculum sessions, structured observations of a randomly selected session per school of one curriculum lesson, interviews with the NSPCC trainer(s) and interviews with intervention school staff.

#### Research and provider and roles

In close collaboration with the research team, NSPCC will lead the elaboration and optimisation of the intervention and the production of materials. In the delivery phase, NSPCC will work independently from the research team to train senior leadership and other key school staff in safeguarding to prevent, recognise and respond to gender-based harassment and DRV; to enable them to lead the intervention in their schools; to review school rules and policies to help prevent and respond to gender-based harassment and DRV; and to identify and increase staff presence in ‘hotspots’ for these behaviours. Trained school staff will then implement the school environment and curriculum components, cascading training in safeguarding to all staff.

#### Comparator

The comparator consists of schools randomly allocated to the control group. Control schools will not implement Project Respect, instead continuing with any existing gender, violence or sexual health-related provision. The study will include three additional activities to support all schools taking part: NSPCC will offer safeguarding officers of all schools a support session to prepare them in case the school experiences increased numbers of students seeking support as a result of the research or intervention (this will take place before the baseline surveys in case of such an increase immediately following baseline surveys; the training therefore takes place before randomisation); the research team will provide a short report to intervention and control schools about the prevalence of DRV reported in their schools; and NSPCC will brief its ‘Childline’ telephone helpline staff so that they are aware of the project in case the research or intervention results in students contacting them. While these activities mean the experience of control schools will differ slightly from treatment as usual, we feel this measured response is essential to fulfil our duty of care to trial participants while not excessively distorting the nature of the comparator. The nature of the comparator will be assessed by examining the sexual health education provision in and around control schools at baseline.

### Outcome measures

In the pilot RCT, the primary outcome will be whether progression to a phase III RCT is justified in terms of the pre-specified criteria listed in research question 1. The pilot RCT will also determine which of two existing DRV scales will be used to measure the primary outcomes of DRV victimisation and perpetration in a phase III trial.

All measures of primary and secondary outcomes and mediators that would be examined in a phase III RCT will also be assessed for reliability in this pilot.

The twin primary outcomes in a phase III RCT would be binary measures of DRV victimisation and perpetration, measured using self-reports rather than via routine data. This is because most experiences of DRV will not result in notifications to the school, police or NHS [43] and our intervention is likely to increase rates of such notifications with the risk of ascertainment bias. While our intervention might also result in increased self-reports, this reporting bias will be minimised by use of a validated and reliable measure comprising items focused on specific behaviours. As there is currently no clear evidence as to whether the SD or CADRI-s measure is the optimal scale to assess DRV victimisation/perpetration in this population, we will adapt and test these measures in this pilot to determine which is most suitable in the UK context.

The SD measure of dating violence is based on self-reported perpetration and victimisation of psychological abuse and of physical and sexual violence in the previous year. Participants are asked ‘How often has anyone that you have ever been on a date with done the following things to you?’ Response options range 0–3, indicating frequency. Items are summed and then recoded 0–3 indicating overall degree of abuse. Psychological abuse is assessed in terms of 14 acts (Cronbach’s alpha = 0.91 for victimisation and 0.89 for perpetration) [47, 60]. Physical and sexual violence are assessed in terms of 18 acts, of which 6 indicate serious physical violence and 2 indicate forced sexual acts (Cronbach’s alphas for perpetration of moderate physical violence = 0.92, for severe physical violence = 0.89 and for sexual violence = 0.86). For victimisation, Cronbach’s alphas are respectively 0.90, 0.86 and 0.74 [47]. The SD measure is one of the most commonly used in research on adolescent dating violence [61] and correlates with poor mental health and various health risk behaviours including other forms of youth violence and substance use [23, 62, 63]. Reliability has been examined in multiple studies of adolescents, but not in the UK to date. We will add introductory text to clarify our interest in both on- and off-line behaviours. Our primary outcome will examine categorical measures of DRV perpetration and victimisation, while secondary outcomes will examine each subscale.

The CADRI measure comprises 92 items assessing DRV victimisation and perpetration over the past year. Subscales cover emotional abuse, relational abuse, controlling behaviours, physical violence and non-consensual sexual activities. Items are rated on a 4-point scale according to frequency, allowing generation of a binary measure of prevalence or a quantitative measure of frequency created from the summed score divided by the number of items. Research has found that DRV as measured via the CADRI scale is correlated during adolescence with early sexual debut, unsafe sex, violence and suicidal ideation [64]. The CADRI instrument has been used in research with young people in the USA, Canada [65, 66] and Spain [67], but not in the UK to date.

The use of the CADRI measure within trials is problematic due to its length. A short 10-item version of the CADRI, the CADRI-s, has been developed and piloted among school-based samples of students in 9th–12th grade and in at-risk samples in Canada. The new measure was found to be slightly less sensitive than the full questionnaire but to have good reliability, fit and convergent validity with the full measure [68]. We plan to further assess this short version. We will modify the scale by adding text clarifying our interest in both on- and off-line behaviours and adding two items from the original CADRI measure to assess experience of controlling behaviours. The developers of the SD and CADRI-s have permitted our use and modification of these measures. We propose to use the pilot RCT to refine the two existing measures, cognitively test these to inform further refinements and then pilot the measures and assess completion rates, inter-item reliability (using Cronbach’s and ordinal alphas) and fit (using confirmatory factor analysis).

Informed by our theory of change, secondary outcomes in a phase III RCT will include the following, which we will assess for reliability in this pilot trial:

- DRV frequency of victimisation and perpetration (using the SD and CADRI-s measures).
- Short Warwick-Edinburgh Mental Well-Being Scale (SWEMWBS). This is a 7-item scale designed to capture a broad concept of positive emotional well-being including psychological functioning, cognitive-evaluative dimensions and affective-emotional aspects [69]. Items are rated on a 5-point scale: none of the time, rarely, some of the time, often, or all of the time. Responses are scored and aggregated to form a ‘well-being index’ with a higher score representing greater well-being [69].
- Paediatric quality of life inventory (PedsQL) version 4.0. This is used to assess overall quality of life. The 23-item PedsQL [70] has been shown to be a reliable and valid measure of quality of life in normative adolescent populations. It consists of 23 items representing 5 functional domains—physical, emotional, social, school and well-being—and yields a total score, two summary scores for ‘physical health’ and ‘psychosocial health’, and three subscale scores for ‘emotional’, ‘social’ and ‘school’ functioning.
- Sexual harassment. Two new items measuring experience of sexual harassment (1) overall and (2) in school, drawing on a widely accepted definition of what constitutes sexual harassment [71].
- Strengths and Difficulties Questionnaire (SDQ). This is a brief, validated instrument for detecting behavioural, emotional and peer problems and pro-social strengths in children and adolescents. It comprises 25 items across five scales assessing emotional symptoms, conduct problems, hyperactivity/inattention, peer relationship problems and prosocial behaviour. A higher total problems score indicates greater problems [72].
- Self-reported sexual health. We will examine pregnancy and unintended pregnancy (initiation of pregnancy for boys) and sexually transmitted infections, age of sexual debut, partner numbers, and use of contraception at first and last sex using measures from previous RCTs [73, 74].
- Self-reported use of primary care, accident and emergency, other service in past 12 months.
- Self-reported contact with police [75].
- School attendance and educational attainment via routine school-level data on half-days absent and General Certificate of Secondary Education (English secondary school qualification) performance for the trial cohorts.

Informed by the intervention’s theory of change, we will also examine the following mediators (to be assessed for reliability in this pilot trial):

- Social norms and gender stereotyping. We will use a modified version of a multi-item subscale developed by Foshee [23] measuring acceptance of prescribed norms (acceptance of dating violence under certain circumstances) using a 4-point Likert scale format, and adapt these items to measure injunctive norms (beliefs about others’ attitudes towards dating violence). Items are averaged to create a composite score [23]. We will use a modified version of items used by Cook-Craig et al. to measure descriptive norms (beliefs about whether DRV is common) [76]. We will measure gender stereotyping using a modified version of the 16-item Attitudes Towards Women Scale, which has high reliability and uses a 4-point Likert scale format [77]. We will adapt these items to measure injunctive norms (beliefs about others’ attitudes towards gender stereotypes).
- Self-reported awareness of services, and help seeking for victims and perpetrators. We will assess these via existing single-item self-report measures [23].
- Communication and anger management. We will assess these using the Modified Sexual Communication Survey (MSCS) and SDQ respectively. MSCS measures open sexual communication with a current or potential partner [78]. The scale includes 21 eight-point Likert scale items examining frequency and has excellent reliability [79, 80].
- Dating violence knowledge. This will be measured using a modified version of a reliable multi-item scale involving true/false questions on help-seeking and definitions [40].
- Downloading and use of the ‘Circle of 6’ app will be measured by a new single-item measure.

To ensure student surveys are age-appropriate, items with sensitive sexual content will be excluded at baseline but included at 16-month follow-up.

#### Economic outcome measures

In this pilot study, the aims of the economic evaluation component are to plan the economic evaluation that would accompany a phase III RCT, identify sources of data and determine how best to collect these. We will undertake a detailed cost analysis of the intervention; collect resource use data and examine response rates and data quality; use the process evaluation to identify any unanticipated costs to students, schools and NSPCC and to consider ways of maximising responses to economic data collection; identify unit costs for the cost components; and review additional literature to identify any new potential sources of data to model long-term costs and outcomes.

In a phase III RCT, the primary economic evaluation would take the form of a within-trial cost-utility analysis, with health outcomes expressed in terms of quality-adjusted life-years (QALYs). Changes in health-related quality of life would be measured primarily from study participants’ perspectives with a secondary analysis examining teacher outcomes. The Child Health Utility (CHU) 9D measure [81] would be used to assess students’ health-related quality of life and the 12-item Short Form Health Survey (SF-12) would be used for this purpose for teachers [82]. In the pilot RCT, we will assess the measures used for this analysis by collecting data on them at baseline and follow-up. The CHU-9 is a validated age-appropriate measure that was explicitly developed using children’s input and has been suggested to be more appropriate and function better than other generic health utility measures for children and adolescents [83]. In a phase III RCT, student and teacher utility values would be collected at baseline and subsequent follow-up points using the selected measures, which would then be converted into utility scores suitable for calculating QALYs using published algorithms. In addition, a cost consequence analysis would be presented with further outcomes. The time horizon would capture costs and outcomes within the trial. In terms of costs, we would present the base-case cost-effectiveness estimate from a public sector perspective, as recommended by the National Institute for Health and Care Excellence’s public health methods guidance. Given that Project Respect will be delivered by a charity, our costing perspective would also be extended to include the voluntary sector.

### Assessment and follow up

Baseline surveys will be conducted before randomisation with two cohorts of students, one nearing the end of year 8 (aged 12–13 years) and one nearing the end of year 9 (aged 13–14 years). Baseline surveys will collect data on socio-demographic variables, pre-hypothesised outcome variables and potential confounders. Where feasible, surveys will be done at the same time of day in all schools. Students will be given an information sheet about the study at least 1 week prior to data collection and an oral description of the study. Students will have the opportunity to ask questions before deciding whether or not to take part. We will be clear about the topics to be explored and the complete anonymity of questionnaire data. Students will then be invited to assent to participate in data collection. All students will be provided with information about school safeguarding officers, other local safeguarding resources (where relevant), a national helpline and other agencies for students experiencing DRV or other forms of abuse. We will also provide students and their parents/guardians with the contact details for the research team to report any concerns relating to the research. As is conventional with UK trials in secondary schools, including trials of sexual health and violence prevention interventions [73, 74, 84], students’ parents/guardians will also be sent a detailed information sheet at least 1 week prior to data collection. They will be asked to contact the school or research team should they have questions or should they wish for their child not to take part. A sample of the information sheets and consent forms used for the study are provided in Additional file 2.

Given the particularly sensitive nature of DRV, we will pilot the use of tablet-based CASI surveys to maximise student privacy and optimise the quality of the data collected. Students will complete surveys confidentially and anonymously with researchers present to explain data collection and support participants where necessary. Teaching staff will be present but will remain at the front of the classroom, helping to maintain order but unable to read student responses. During optimisation, we will ask students about the acceptability of this approach.

We will survey absent students by leaving paper questionnaires and stamped addressed envelopes with their schools. When we conduct follow-up surveys 16 months post-baseline, with students who are near the beginning of years 10 and 11 (aged 14–15 and 15–16 years, respectively), we will collect self-report data on intervention participation, outcomes and potential mediators. Fieldworkers will be blind to school allocation. Based on past experience [84], in the pilot, we anticipate 95% baseline survey participation and 90% at follow-up. We will also conduct online staff surveys at baseline and 16 months post-baseline for the economic and process evaluations.

### Process evaluation

An integral process evaluation, informed by existing frameworks [85–87], has three purposes: first, to examine intervention feasibility, fidelity, reach and acceptability; second, to assess provision of sexual health services and violence prevention in and around control schools; and third, to explore context and potential mechanisms of action, as well as potential unintended effects, in order to refine the intervention’s theory of change and the intervention methods.

#### Intervention feasibility, fidelity, reach and acceptability

In addition to assessing the ‘progression criteria’ outlined in the study’s research question 1 relating to intervention feasibility and acceptability, we will also examine reach and how it varies by student and school characteristics. Data on these outcomes will be collected via: audio-recording of all NSPCC and school-delivered training (fidelity); logbooks completed by teaching staff delivering all curriculum sessions (feasibility, fidelity, costs); structured observations of a randomly selected session per school of one curriculum lesson (fidelity); student surveys (reach, acceptability); staff survey (reach, acceptability of training and intervention overall); interviews with the NSPCC trainer(s) (feasibility, fidelity); interviews with four staff per intervention school, purposively sampled by seniority and which intervention component(s) they are involved in (acceptability, fidelity); interviews with two parents per intervention school, purposively sampled by age and sex of their child (acceptability); and interviews with eight students per intervention school, purposively sampled by year 9/10, sex and involvement in a student-led campaign as part of the intervention delivery (acceptability).

Fidelity will be assessed quantitatively against tick-box quality metrics. For example, each training and curriculum session will be assessed against session-specific quality metrics relating to the topics covered, the exercises used and opportunities for discussion. After the intervention is fully elaborated, the investigators will finalise the fidelity metrics based on the intervention and will ask the Study Steering Committee (SSC) to approve these prior to their use in the process evaluation.

Trained researchers will conduct interviews in private rooms, guided by semi-structured interview guides. Although the qualitative research will not aim to explore students’ personal experiences of sex, relationships, or DRV, disclosures of abuse may occur. In focus groups, we will instruct participants not to disclose any experiences of abuse during the group discussion since we cannot guarantee that all participants would keep this information confidential. All focus groups will be conducted by researchers who have been trained to steer group discussions away from potential disclosures. We will, however, provide the opportunity for participants to speak with the researcher in private after the focus group if they would like help with any issues they are facing. If disclosures of sexual intercourse before age 13 years or of any other abuse occur during qualitative data collection, the researcher will establish whether the reported abuse meets our criteria for referral. If it does, the researcher will inform the student that she or he must report this to the school safeguarding officer. We have defined categories of harm warranting such responses with the advice of a social worker specialising in child protection and in collaboration with NSPCC (see the ‘Ethical issues’ section, below). We will consult with school safeguarding officers in advance to ensure this process is compatible with school policies.

Interviews will be audio-recorded and transcribed in full. Drawing on May’s theory of implementation [86], qualitative research will assess how implementation is influenced by NSPCC and school staffs’ perceptions as to the intervention’s potential workability and integration within the school system, possession of the required norms and relationships to underpin implementation, shared commitment to enact the complex intervention and continuous contributions that are sustained in time and space.

#### Provision in control schools

We will examine sexual health provision in and around control schools to describe our comparator. Data on this will be collected via staff and student surveys; interviews with two staff members per control school, selected purposively by seniority; and four students per control school, selected purposively by year 9/10 and sex.

#### Context and mechanisms of action

Informed by realist approaches [88, 89], using qualitative methods we will aim to explore potential intervention mechanisms and how these interact with contextual factors to enable outcomes, including mechanisms that might give rise to unintended, potentially harmful consequences. We will also explore how potential mechanisms of action might vary with school context and student characteristics, in order to refine and optimise the intervention’s theory of change and intervention methods. Data on context and mechanisms will be collected via interviews with NSPCC trainers, student and staff surveys and interviews with four staff and eight students per intervention school (purposively sampled as described above). Our quantitative research will pilot mediator analyses, as discussed in the next section.

### Approach to data analysis

In the pilot RCT, our primary analysis will determine whether criteria for progression to a phase III RCT are met. Descriptive statistics on fidelity will draw on audio-recordings of training, logbooks completed by teaching staff and structured observations of curriculum lessons. Acceptability will be assessed through student and staff surveys. Recruitment and response rates will be reported in a flow chart and used to refine our power calculation. Pilot RCT analyses will also assess which of our indicative primary outcomes is sufficiently reliable to use within a phase III RCT, assessing response rates, inter-item reliability (using Cronbach’s and ordinal alphas) and fit (using confirmatory factor analysis). In-line with our approach in a previous pilot trial, we will prioritise completion rates and inter-item reliability when judging between measures [84]. We will set the threshold for acceptable reliability at a Cronbach’s alpha of 0.70 or higher. If both measures perform well on this, we will choose the CADRI-s for use in a phase III RCT since this is the more established measure. If neither performs well, we will not progress to phase III without first identifying and piloting alternative measures.

Although the pilot RCT will be underpowered to determine an ICC and prevalence among the comparator of DRV, it will enable a more qualitative assessment of whether estimates derived from North American studies seem to be appropriate for schools in England.

Data from the process evaluation will be analysed to describe provision of violence prevention and sexual health-related activities in and around study schools, contextual influences on intervention feasibility and acceptability and potential mechanisms of benefits and unintended impacts to refine the intervention’s theory of change. Qualitative data will be subject to thematic content analysis using techniques drawn from grounded theory such as in vivo/axial codes and constant comparison [90]. As well as deriving themes inductively from the data, we will also use realist approaches to evaluation [89] and May’s implementation theory [86] to inform analyses, identifying characteristics of the intervention, providers and settings which promote or hinder implementation or which might interact with intervention mechanisms to enable outcomes. Qualitative research will develop hypotheses which will be tested in exploratory quantitative analyses where data allow.

The economic evaluation feasibility component of the study will pilot measures assessing quality of life and assess the feasibility of methods to be used within a full RCT. We will also pilot the primary intention-to-treat analyses of outcomes which will use repeat cross-sectional data as would be done within a phase III RCT, as well as secondary, moderator and mediator analyses. In a phase III RCT, moderator analysis would be conducted to examine how effects vary by student socioeconomic status, sex and ethnicity and by school IDACI and value-added academic attainment. Mediator analysis would examine whether intervention effects on mediators might explain effects on our primary outcomes using established methods [91]. All such analyses will be underpowered in this pilot RCT but will be piloted to refine methods.

### Protecting against bias

The aim of this study is to pilot the intervention and RCT methods, not to estimate intervention effects. However, we will pilot methods aimed at minimising bias. The research team and the intervention delivery team will be separately managed. We will aim to maximise response rates to reduce non-response and attrition bias, for example by following up with schools to collect surveys from those individuals not present during survey sessions. Response rates and qualitative data will be analysed to refine data collection methods prior to a phase III RCT.

### Ethical issues

Ethical approval for the study has been obtained from the London School of Hygiene and Tropical Medicine Ethics Committee and the NSPCC Research Ethics Committee. All work will be carried out in accordance with guidelines laid down by the Economic and Social Research Council, the Data Protection Act 1998 and the latest Directive on Good Clinical Practice (2005/28/EC). Any member of the research/fieldwork team visiting a school to conduct unsupervised research with a student will be required to have a full Disclosure and Barring Services check. Quantitative and qualitative data will be managed by project staff using secure data management systems and stored anonymously. Quantitative data will be managed by LSHTM, an accredited CTU. All data will be stored in password-protected folders. The names used in qualitative data will be replaced with pseudonyms in interview transcripts. In reporting the results of the qualitative research, care will be taken to use quotations that do not reveal the identity of respondents. In line with Medical Research Council guidance on personal information in medical research, we will retain all research data for 20 years after the end of the study [92]. This is to allow secondary analyses and further research to take place, and to allow any queries or concerns about the conduct of the study to be addressed. In order to maintain the accessibility of the data, the files will be refreshed annually and upgraded if required.

Any disclosures of abuse that meet the criteria for a serious adverse event (SAE) or suspected unexpected serious adverse reaction (SUSAR; defined as an unexpected SAE) will be reported in anonymised form to the SSC (which, because this is a pilot and not a phase III RCT, will undertake data monitoring and ethics duties) and to the LSHTM and NSPCC ethics committees. Reporting will be in real time if the event might plausibly have been caused by the intervention or research. Any other SAEs and SUSARs will be reported to these committees annually. Reporting will include the type of event, circumstances, extent of any possible connection with intervention or research activities and outcome of the response.

### Research governance

#### Study registration

The pilot RCT has was registered on 8th June 2017 with the ISRCTN registry (ISRCTN 65324176). 10.1186/ISRCTN65324176

#### Study management

The principal investigator (PI), Chris Bonell (CB), will have overall responsibility for the conduct of the study. The day-to-day management of the RCT will be coordinated by Rebecca Meiksin (RM), the study manager based at LSHTM. The following governance structures will be instituted: a study executive group (SEG) where the PI (CB) will chair fortnightly meetings with the study manager (RM), statistician Elizabeth Allen (EA) and, where appropriate, CTU and fieldwork staff; a study investigators’ group (SIG) chaired by CB which includes all co-investigators and members of the SEG and which will meet monthly during the early stages of the research (months 1–6), then every 3 months thereafter; and an SSC which will meet three times throughout the life of the project to advise on the conduct and progress of the study and on relevant practice and policy issues. The SSC will also undertake data monitoring and ethics duties. The project will employ standardised research protocols and pre-specified progression criteria, which have been agreed and will be monitored by the SIG and SSC.

### Consultation with public and stakeholders

The intervention will be elaborated and optimised by the NSPCC and the study team working with the ALPHA young people’s research advisory group, policy stakeholders and school staff, as well as with young people recruited via an organisation that provides support to survivors of sexual abuse to ensure the intervention and evaluation are sensitive to the needs and preferences of young people directly affected by DRV. School staff and young people from the ALPHA group will also be consulted on research methods at the beginning of the study on recruitment, assent/consent materials, refinements of DRV scales and survey methods and strategies for increasing retention; and at the end of the study on RCT and intervention refinement and knowledge transfer. We will also convene two meetings with policy stakeholders, including representatives from the Association for Young People’s Health, the Department for Education, the Department of Health, Public Health England, the Personal, Health, Social and Economic PSHE Association and an organisation providing support services to survivors of sexual abuse. The meetings will take place at the start to build support for the study and ensure it is policy-relevant, and near the end to inform preparations for a full RCT and knowledge transfer.

## Discussion

To our knowledge, this will be the first trial of an intervention that aims to reduce DRV among adolescents in the UK. Drawing on evidence from existing reviews and from promising interventions trialled in the USA, and underpinned by behavioural change theory, the Project Respect intervention will be optimised for the UK through work with students, school staff and policy stakeholders. We will pilot baseline and follow-up CASI surveys, assessing feasibility and acceptability of the research methods and determining whether the SD or CADRI-s scale is optimal for assessing the primary outcome measures of DRV perpetration and victimisation in a phase III RCT.

Informed by realist methods, the integral process evaluation will use qualitative methods to explore potential intervention mechanisms and how these interact with contextual factors to elicit both intended and unintended outcomes.

Judged against pre-specified criteria, findings from this pilot cluster RCT will determine whether progression to a phase III RCT is justified. If it is, learning from this pilot will inform refinement of the intervention, its theory of change and the research methods for a full-scale trial.

## Recruitment status

Participant enrolment for baseline surveys began in June 2017. At the time of submission (May 2018), the optimisation of the intervention and the student and staff baseline surveys have been carried out. Schools are in the process of implementing the intervention and the research team is currently recruiting participants for the process evaluation.

## Additional files

Additional file 1: SPIRIT Checklist. (DOC 121 kb)

Additional file 2: Consent Forms and Information Sheets for interviews with students in intervention schools. These reflect the structure and content of such documents used for the data collection activities conducted throughout the study. Separate Consent Forms and Information Sheets were developed for each recruitment and data collection activity, yielding a total of 44 such documents. For data collection involving students, separate Information Sheets were developed for students and for their parents/guardians. The Consent Forms and Information Sheets not included in this file are available upon request. (ZIP 1209 kb)

## Abbreviations

CASI: Computer-assisted self-interview
CHU-9D: Child Health Utility 9D
CTU: Clinical trials unit
DRV: Dating and relationship violence
ICC: Intra-cluster correlation coefficient
IDACI: Income deprivation affecting children index
LSHTM: London School of Hygiene & Tropical Medicine
NSPCC: National Society for the Prevention of Cruelty to Children
PedsQL: Paediatric quality of life inventory
PSHE: Personal, Health, Social and Economic
QALYs: Quality-adjusted life-years
RCT: Randomised controlled trial
SAE: Serious adverse event
SF-12: 12-Item Short Form Health Survey
SPIRIT: Standard Protocol Items: Recommendations for Intervention Trials
SSC: Study Steering Committee
SUSAR: Suspected unexpected serious adverse reaction
SWEMWBS: Short Warwick-Edinburgh Mental Well-Being Scale
TIDieR: Template for Intervention Description and Replication
