# Supplementary material for: Protocol for pilot cluster RCT of project respect: a school-based intervention to prevent dating and relationship violence and address health inequalities among young people
Source: Pilot Feasibility Stud. 2019 Jan 22;5:13. doi: 10.1186/s40814-019-0391-z (PMC6341537; doi:10.1186/s40814-019-0391-z)
Supplement: Supplementary file 2 — Consent Forms and Information Sheets for interviews with students in intervention schools. These reflect the structure and content of such documents used for the data collection activities conducted throughout the study. Separate Consent Forms and Information Sheets were developed for each recruitment and data collection activity, yielding a total of 44 such documents. For data collection involving students, separate Information Sheets were developed for students and for their parents/guardians. The Consent Forms and Information Sheets not included in this file are available upon request. (ZIP 1209 kb) [file 40814_2019_391_MOESM2_ESM.zip › Add. File 4_Interv school interview_Parent info sheetR1.doc]

**Information Sheet for Parents/Guardians**

We are researchers working at your child’s school. We are evaluating “Project Respect,” a programme being delivered in some English secondary schools which aims to prevent violence within young people’s dating and relationships. The programme includes activities in the school and information sent to parents which aim to help keep young people safe.

As part of our research, we are speaking with students, parents and school staff to find out about their views on and experiences with the programme. We would like to invite your child to take part in an interview. The school has chosen your child for the research team to invite for an interview based on their year group, their gender and their involvement in Project Respect. Your child has not been selected because he or she is perceived to be at particular risk or for any other reasons. The interview will take no more than 1 hour. Your child will only be interviewed if she or he agrees. They will receive information about the interview and be able to ask questions before they decide.

We will not ask your child about their own experiences of dating, relationships or violence. If your child agrees to take part, a researcher will ask them about:

- Themselves and how long they have been at the school
- Life at the school in general, including dating violence and harassment at the school
- Their experience of the “Project Respect” programme
- Their views on the “Project Respect” programme, its activities and its impact in the school

Some students might find some questions sensitive or feel upset, particularly if they have experienced abuse. All students who are invited to take part will receive information about support resources both within and outside of the school, should they wish to speak with someone about any issues they (or a friend) are going through.

What your child tells us will be used to try and improve schools and young people’s health, and their answers will be completely confidential. No one except the research team will find out about what they say. However, if your child tells us that they or another student are at risk of very serious harm or had sex before age 13, we will need to tell someone in charge of safeguarding at the school so that they can help them.

We would like to audio-record the interview and then produce a written record of what was said. Neither will include your child’s name. When we write articles or reports about what we find, we might include quotes from the interview but will not include your child’s name or any information that could identify them or their school.

If you are happy for your child to participate, you do NOT need to do anything. If you are not happy for them to take part, if you have any questions, or if you have any concerns or complaints about the research, please contact the study manager, XXXX, by telephone (XXXX) or via email (XXXX).

If you prefer to speak with someone outside of the research team, you can tell the school directly that you do not want your child to participate, or share any concerns or complaints about the research, by contacting [school’s study liaison staff member] by telephone (XXXX) or email (XXXX).

If you have any concerns about any child in the school, including if you are concerned that they are experiencing abuse or neglect or that they are harming someone else, you may contact the school’s safeguarding lead, XXXX, at XXXX. If you seek their support, the safeguarding lead will hold an initial meeting with you to determine the nature of your concern and the appropriate response.

If you are concerned about a child for any reason, you can also call the **NSPCC helpline** 24 hours a day for advice or to share your concerns, anonymously if you wish, on **0808 800 5000**.

Below is some information about other organisations that may be able to help you with issues you or someone you know might be going through:

- **[local safeguarding referral utilised by the school]:** Local service available for any concerns related to child safeguarding. Call XXXX.
- **The Samaritans**: Someone to talk to, available 24 hours a day for confidential, non-judgmental support. Call 116 123 or visit [www.samaritans.org](http://www.samaritans.org/)
- **National Domestic Violence Helpline**: National service available 24 hours a day for women experiencing domestic violence, and for their family, friends, colleagues and others calling on their behalf. Call 0808 2000 247.
- **Switchboard LGBT+ Helpline**: Providing information, support and referral services for lesbians, gay men and bisexual and trans people, and anyone considering issues around their sexuality or gender identity. Call 0300 330 0630 or visit http://switchboard.lgbt/help/
- **Mind**: Offering advice and support for anyone experiencing a mental health problem. Call 0300 123 3393 or visit [www.mind.org.uk](http://www.mind.org.uk/)
- **Young Minds Parents Helpline:** Provides confidential advice for parents concerned about a child’s behaviour, emotional wellbeing or mental health condition. Call 0808 802 5544 or visit [www.youngminds.org.uk](http://www.youngminds.org.uk/)
- **Rape Crisis:** Provides information on nearest services for people who have experienced sexual violence. Call 0808 802 9999 or visit their website [www.rapecrisis.org.uk](http://www.rapecrisis.org.uk/)

Many thanks for your time,

Professor Chris Bonell (London School of Hygiene and Tropical Medicine)
