# Supplementary material for: Protocol for pilot cluster RCT of project respect: a school-based intervention to prevent dating and relationship violence and address health inequalities among young people
Source: Pilot Feasibility Stud. 2019 Jan 22;5:13. doi: 10.1186/s40814-019-0391-z (PMC6341537; doi:10.1186/s40814-019-0391-z)
Supplement: Supplementary file 2 — Consent Forms and Information Sheets for interviews with students in intervention schools. These reflect the structure and content of such documents used for the data collection activities conducted throughout the study. Separate Consent Forms and Information Sheets were developed for each recruitment and data collection activity, yielding a total of 44 such documents. For data collection involving students, separate Information Sheets were developed for students and for their parents/guardians. The Consent Forms and Information Sheets not included in this file are available upon request. (ZIP 1209 kb) [file 40814_2019_391_MOESM2_ESM.zip › Add. File 3_Interv school interview_Student info sheetR1.doc]

**Information sheet for student interviews**

**What is the research about?**

We are researchers working at your school. We are evaluating “Project Respect,” a programme being delivered in some English secondary schools which aims to prevent violence within young people’s dating and relationships. The programme includes activities in the school and information sent to parents which aim to help keep young people safe.

As part of our research, we are speaking with students, parents and school staff to find out about their views on and experiences with the programme. We would like to invite you to take part in an interview. The school has chosen you for the research team to invite for an interview based on your year group, your gender and your involvement in Project Respect. You have not been selected because you are perceived to be at particular risk or for any other reasons.

**What is an interview?**

An interview involves you talking with a researcher. This will take no more than 1 hour.

**What will I be asked about?**

We will not ask you about your own experiences of dating, relationships or violence. If you agree to take part, the researcher will ask you about:

- Yourself and how long you’ve been at the school
- Life at the school in general, including dating violence and harassment at the school
- Your experience of the “Project Respect” programme
- Your views on the “Project Respect” programme, its activities and its impact in the school

**Do I have to take part?**

No, you do not have to take part. It is completely up to you to decide. If you do choose to take part, and you don’t want to answer a particular question, you can skip it. If you feel uncomfortable, find it difficult to talk about anything or change your mind for any reason, we can stop the interview at any point, with no negative consequences.

**What support is available?**

Some students might find some of the questions sensitive or feel upset, particularly if they have experienced abuse.

If you wish to speak with someone about any issue you or someone you know are going through, there is a list at the end of this information sheet of people and organisations you can contact for support, both inside and outside your school.

If you would like to talk with a researcher privately before or after the interview about any concerns or issues you would like help with, we will be happy to talk with you and to connect you with someone at your school or the NSPCC (a charity that works with young people) who is in charge of safeguarding and can help you.

**What will happen with the information from the interview?**

We would like to audio-record the interview and then produce a written record of what was said. Neither will include your name. Your parents or guardians, teachers and the police will not be told what was said during the interview.

When we write articles or reports about what we learn from the research, we might include quotes from the interview. However, we will not include any information that could identify you or your school.

Anything you say will be kept confidential. However, if you tell us that you or another student are at risk of very serious harm or had sex before age 13, we will need to tell someone at the school who is in charge of safeguarding so that they can help.  If this happens, we will first discuss it with you.

**What happens next, if I wish to take part?**

If you are happy to take part in the interview, you do NOT need to do anything. We will follow up to arrange a time that is convenient for you for the interview.

**What if I do not wish to take part, or if I have questions, concerns or complaints?**

The study manager’s contact information is:

Name: XXXX

Telephone: XXXX

Email: XXXX

You can contact the study manager if:

- You have any questions
- You are not happy to take part
- You have any concerns or complaints about the research

If you prefer to speak with someone outside of the research team, you can tell the school directly that you do not want to take part, or share any concerns or complaints about the research, by contacting:

Name: [school’s study liaison staff member]

Telephone: XXXX

Email: XXXX

**Details of support available**

***Within the school***

If you would like to talk to someone at your school about how you are feeling or any issues you or someone you know are going through, XXXX is in charge of safeguarding at your school and can help you. For example, this might include if you or someone you know are experiencing abuse or neglect or if you are concerned that someone you know is harming someone else. If you ask for their help, the safeguarding lead will meet with you to find out more about your concern and how to respond.

***Outside of the school***

If you need help outside of school time you can contact [local safeguarding referral utilised by the school if available over school holidays] by calling XXXX.

You can also call the **NSPCC Childline** on **0800 1111** if you want help or support with any issue you are going through, no matter how big or how small.

Below is some information on other organisations that may be able to help with any issues you or someone you know might be going through:

- **The Samaritans**: Someone to talk to, available 24 hours a day for confidential, non-judgmental support. Call 116 123 or visit [www.samaritans.org](http://www.samaritans.org/)
- **Switchboard LGBT+ Helpline**: Providing information, support and referral services for lesbians, gay men and bisexual and trans people, and anyone considering issues around their sexuality or gender identity. Call 0300 330 0630 or visit http://switchboard.lgbt/help/
- **Mind**: Offering advice and support for anyone experiencing a mental health problem. Call 0300 123 3393 or visit [www.mind.org.uk](http://www.mind.org.uk/)
- **Rape Crisis:** Provides information on nearest services for people who have experienced sexual violence. Call 0808 802 9999 or visit their website [www.rapecrisis.org.uk](http://www.rapecrisis.org.uk/)

Many thanks for your time,

Professor Chris Bonell (London School of Hygiene and Tropical Medicine)
