# Supplementary material for: Protocol for pilot cluster RCT of project respect: a school-based intervention to prevent dating and relationship violence and address health inequalities among young people
Source: Pilot Feasibility Stud. 2019 Jan 22;5:13. doi: 10.1186/s40814-019-0391-z (PMC6341537; doi:10.1186/s40814-019-0391-z)
Supplement: Supplementary file 2 — Consent Forms and Information Sheets for interviews with students in intervention schools. These reflect the structure and content of such documents used for the data collection activities conducted throughout the study. Separate Consent Forms and Information Sheets were developed for each recruitment and data collection activity, yielding a total of 44 such documents. For data collection involving students, separate Information Sheets were developed for students and for their parents/guardians. The Consent Forms and Information Sheets not included in this file are available upon request. (ZIP 1209 kb) [file 40814_2019_391_MOESM2_ESM.zip › Add. File 2_Interv school student interview_Consent formR1.doc]

**Consent form for student interviews**

**What is the research about?**

We are researchers working at your school. We are evaluating “Project Respect,” a programme being delivered in some English secondary schools which aims to prevent violence within young people’s dating and relationships. The programme includes activities in the school and information sent to parents which aim to help keep young people safe.

As part of our research, we are speaking with students, parents and school staff to find out about their views on and experiences with the programme. We would like to invite you to take part in an interview. The school has chosen you for the research team to invite for an interview based on your year group, your gender and your involvement in Project Respect. You have not been selected because you are perceived to be at particular risk or for any other reasons.

Please read this sheet. If you’re willing to take part, please fill in the box below. This is to show us that you understand what this involves and are happy to take part.

**What is an interview?**

An interview involves you talking with a researcher. This will take no more than 1 hour.

**What will I be asked about?**

We will not ask you about your own experiences of dating, relationships or violence. If you agree to take part, a researcher will ask you about:

- Yourself and how long you’ve been at the school
- Life at the school in general, including dating violence and harassment at the school
- Your experience of the Project Respect programme
- Your views on the “Project Respect” programme, its activities and its impact in the school

**Do I have to take part?**

No, you do not have to take part. It is completely up to you to decide. If you do choose to take part, and you don’t want to answer a particular question, you can skip it. You can also stop the interview at any point, with no negative consequences.

**What will happen with the information from the interview?**

We would like to audio-record the interview and then produce a written record of what was said. Neither will include your name. Your parents or guardians, teachers and the police will not be told what was said during the interview.

When we write articles or reports about what we learn from the research, we might include quotes from the interview. However, we will not include any information that could identify you or your school.

Anything you say will be kept confidential. However, if you tell us that you or another student are at risk of very serious harm or had sex before age 13, we will need to tell someone at the school who is in charge of safeguarding so that they can help.  If this happens, we will first discuss it with you.

***If you have any questions the researcher will be happy to answer them. If you’re happy to take part, please fill in the consent box below, ticking the tick-boxes to indicate you agree.***

**Full Name	……………………………………………………..…………..**

**I have read the information above.**

**I understand that I can choose to take part or not.**

**I understand that I can stop taking part at any time.**

🞏 **I agree to take part in this interview**

🞏 **I agree to have this interview audio-recorded**

**Signed ……………………………. Date …………………………………**
